# Progressive Brain Damage, Synaptic Reorganization and NMDA Activation in a Model of Epileptogenic Cortical Dysplasia

**DOI:** 10.1371/journal.pone.0089898

**Published:** 2014-02-27

**Authors:** Francesca Colciaghi, Adele Finardi, Paola Nobili, Denise Locatelli, Giada Spigolon, Giorgio Stefano Battaglia

**Affiliations:** 1 Molecular Neuroanatomy and Pathogenesis Unit, IRCCS Neurological Institute “C. Besta”, Milano, Italy; 2 Neuroscience Institute Cavalieri Ottolenghi (NICO), University of Torino, Orbassano (Torino), Italy; Università di Trento, Italy

## Abstract

Whether severe epilepsy could be a progressive disorder remains as yet unresolved. We previously demonstrated in a rat model of acquired focal cortical dysplasia, the methylazoxymethanol/pilocarpine - MAM/pilocarpine - rats, that the occurrence of status epilepticus (SE) and subsequent seizures fostered a pathologic process capable of modifying the morphology of cortical pyramidal neurons and NMDA receptor expression/localization. We have here extended our analysis by evaluating neocortical and hippocampal changes in MAM/pilocarpine rats at different epilepsy stages, from few days after onset up to six months of chronic epilepsy. Our findings indicate that the process triggered by SE and subsequent seizures in the malformed brain **i**) is steadily progressive, deeply altering neocortical and hippocampal morphology, with atrophy of neocortex and CA regions and progressive increase of granule cell layer dispersion; **ii**) changes dramatically the fine morphology of neurons in neocortex and hippocampus, by increasing cell size and decreasing both dendrite arborization and spine density; **iii**) induces reorganization of glutamatergic and GABAergic networks in both neocortex and hippocampus, favoring excitatory *vs* inhibitory input; **iv**) activates NMDA regulatory subunits. Taken together, our data indicate that, at least in experimental models of brain malformations, severe seizure activity, i.e., SE plus recurrent seizures, may lead to a widespread, steadily progressive architectural, neuronal and synaptic reorganization in the brain. They also suggest the mechanistic relevance of glutamate/NMDA hyper-activation in the seizure-related brain pathologic plasticity.

## Introduction

The question whether repeated seizures might be associated with progressive alterations of the brain has been long debated and as yet unresolved [1]. Even though recent MRI studies showed that human pharmaco-resistant temporal lobe epilepsy (TLE) was associated with progressive and diffuse cortical atrophy, likely representing seizure-induced damage [2], the causal relationship between seizure activity and brain damage is still controversial [3]. Likewise, the sequence of events possibly leading to disease progression in drug-resistant epileptic patients and experimental models remains elusive [4]–[6].

Focal cortical dysplasia (FCD) is a malformation of cortical development frequently associated with severe drug-resistant focal epilepsy [7]–[8]. First introduced by Taylor and colleagues [9], the term FCD now identifies restricted or diffuse abnormalities of the cortical structure in patients undergoing surgery for particularly severe epilepsy [8], [10]–[12]. Cortical specimens from FCD patients showed increased propensity to generate epileptiform activity, possibly due to enlarged dysmorphic neurons acting as epileptic generators [13], and/or to excessive glutamatergic synaptic input associated with reduced inhibition. Data from surgical specimens of epileptic FCD patients demonstrated increased expression levels of NR2A/B subunits of the NMDA (N-Methyl-D-Aspartate) receptor [14]–[18] and associated PSD95 protein [19]. In addition, epileptogenic activities were sensitive to NR2B-specific inhibitors [20]. On the other hand, reduced mRNA levels of GABA-A (γ-aminobutyric acid) receptor subunit [14] and decreased pre-synaptic release [21] indicated a role in FCD epileptogenesis for abnormalities of GABA-mediated synaptic inhibition. Nevertheless, the precise mechanisms of the intrinsic hyperexcitability in FCD remain to be fully clarified.

Experimental models of cortical dysplasia might be instrumental in exploring mechanisms of epileptogenesis on one side and the possible progression of epilepsy related brain abnormalities on the other [22]–[25]. We recently demonstrated that in a rat model of acquired FCD (MAM/pilocarpine rats) [26] the occurrence of status epilepticus (SE) and spontaneous seizures gives rise to abnormally large cortical pyramidal neurons with neurofilament over-expression and recruitment of NMDA regulatory subunits at the post-synaptic membrane, strictly similar to the hypertrophic/dysmorphic neurons observed in human FCD [10]. The model therefore suggests, at least in cortical pyramidal neurons, the existence of seizure-related synaptic and cellular remodeling (*i.e., pathologic plasticity*) possibly contributing to the process of epileptogenesis.

In the present paper, we further exploited the MAM/pilocarpine model by analyzing epileptic rats at different time-points after epilepsy onset to verify the hypothesis of progressive anatomical and molecular changes over time. The results here reported indicate the presence of a widespread, steadily progressive pathologic process taking place in the epileptogenic malformed brain.

## Materials and Methods

### Ethic statement

Procedures were conducted with care to minimize discomfort and pain to treated rats in compliance with national (D.L.n.116, G.U., Suppl 40, February 18, 1992) and international guidelines and laws (EEC Council Directive 86/609, OJ L 358, 1, December 12, 1987, Guide for the Care and Use of Laboratory Animals, U.S. National Research Council, 1996). The experimental protocol was approved by the Ethics Committee of the “C. Besta” Neurological Institute and by the Italian Ministry of Health (protocol number: BR1/2012).

### MAM and pilocarpine administration

MAM/pilocarpine (MAM-PILO or MP) rats were prepared as described previously [26]. First, to generate offspring with cerebral malformations [27]–[28], pregnant Sprague–Dawley rats (Charles River, Calco, Italy) received 2 methyl-axozymethanol- acetate (MAM) intraperitoneal (i.p.) doses (15 mg/kg maternal body weight, in sterile saline) at the same embryonic day (E15) 12 hours apart.

The pro-convulsant agent pilocarpine (270 mg/kg) was then administered to induce SE in 83 adult MAM rats (250–300 g, ∼3 months old) as previously reported [26]. Thirty minutes before pilocarpine, rats were i.p. injected with N-methylscopolamine (1 mg/kg) for minimizing peripheral cholinergic activation and reducing seizure-induced injuries [29]. In the 8 h period after pilocarpine, at least two researchers monitored continuously the rats, recording and rating pilocarpine-induced symptoms. Death occurring during SE could not be anticipated on the basis of external monitoring. MP rats surviving SE were injected with phenobarbital (20 mg/kg i.p.) 90 minutes after SE to suppress seizures and decrease mortality rate. Care was also undertaken to avoid reduction of body temperature. In the following four days, rats were hydrated subcutaneously with lactate Ringer's solution and fed by the researchers to improve survival and accelerate recovery. Additional 21 MAM rats were pre-treated with 5 mg/kg diazepam (DZP) i.p. 30 min before pilocarpine: they did not experience either SE or epilepsy (MAM-DZP-PILO or MDP) [26].

### SE and seizure assessment

MP rats and MDP controls were continuously video-recorded (during the day of SE induction and for 24 h/day after SE) to monitor the development of SE and detect the onset of spontaneous seizures. The onset of SE was defined by the occurrence of continuous seizure activity (stage 4 or 5 in Racine scale [30]). After epilepsy onset, MP rats were video-monitored every 5 days (6 p.m. to 10 a.m.) up to 6 months to quantify seizure activity. Seizures were graded as follows: stage 0 through 5, as outlined by Racine [30]; stage 6, cluster of multiple stage 5 seizures; stage 7, jumping and running seizures; stage 8, stage 7 plus tonic hindlimb extension and tail rigidity [31]–[32]. Stages 4 to 7 were considered in seizure assessment, stage 8 seizures were never observed during video-monitoring of chronic epileptic rats. The following parameters: i) weight loss >15% or ii) reluctance to eat and drink or iii) inability to move, were defined as humane endpoints dictating the sacrifice of experimental epileptic rats. However, we never observed physical conditions prodding us to euthanize our experimental rats before the established time-points.

Randomly chosen epileptic MP rats were sacrificed at different time points: **i**) 3–5 days, hereafter defined as early-chronic (EC) MP rats (MP-EC, n = 8 rats), **ii**) 3 months (MP-3m: n = 18) and **iii**) 6 months (MP-6m: n = 12) after epilepsy onset, and used for subsequent analysis. MDP rats not experiencing either behavioral SE or seizures were sacrificed 3 or 6-9 months after diazepam-pilocarpine administration and used as controls (hereafter indicated as MDP-3m and MDP-6m). Table 1 reports the number of rats used in the different experimental procedures.

**Table 1:** Study design and number of rats used in the different experiments.

*Experimental group*	*No SE*	*Dead in SE*	*Histology*	*WB*	*Golgi-Cox*	*Dead in chronic*
MDP (n = 21)	21	—				—
3 months			8	6	4	
6–9 months			3			
MP (n = 83)	22	16				7
early chronic			4	4		
3 months			7	6	5	
6 months			8	4		

MDP control rats were sacrificed 3 or 6–9 months after diazepam-pilocarpine administration (respectively MDP-3m and MDP-6m). Randomly chosen epileptic MP rats were sacrificed respectively 3–5 days (early chronic), 3 months and 6 months after epilepsy onset. Seven chronic epileptic MP rats were found dead (three rats, ∼1 month post-SE; two rats, ∼4 months post-SE and two rats, ∼6 months post-SE). MDP, MAM/diazepam/pilocarpine treated rats; MP, MAM/pilocarpine treated rats; WB, western blot; SE, status epilepticus. No SE: rats not experiencing SE.

### Cerebral tissue preparation

For morphologic/morphometric analysis, rats were anesthetized with chloral hydrate (1 ml/100 g body weight of a 4% solution) and perfused with 4% paraformaldehyde in 0.1 M phosphate buffered saline at pH 7.2. After perfusion, brains were removed from the skull, post-fixed overnight, cut with a vibratome (Leica Biosystem, Wetzlar, Germany) into 40 to 50 µm thick coronal sections and collected in serial order. For Golgi-Cox impregnation, 5 MP-3m rats and 4 MDP-3m controls were anesthetized with chloral hydrate and perfused with 0.9% saline (see below). For western blot (WB) analysis, MP rats and MDP controls were sacrificed by decapitation and their brains immediately removed. Cortical heterotopic areas and hippocampi were dissected out from the surrounding cerebral areas under microscopic guidance [26], [33]. At the time of sacrifice, MP epileptic rats were seizure-free for at least 4 hours.

### Morphologic analysis

One series of coronal sections (1 out of 7 sections) was counterstained with 0.1% thionine and the adjacent sections were processed for immunocytochemistry (ICC) or immunofluorescence (IF) as previously described [15]. The following primary antibodies were used: monoclonal anti-SMI 311 against non-phosphorylated neurofilaments (1∶500; Sternberger Monoclonals Inc, Lutherville, MD, USA); monoclonal anti-Neurofilament 200 (NF200, 1∶500; Sigma-Aldrich, Saint Louis, MO, USA); rabbit polyclonal anti-vesicular GABA transporter (VGAT, 1∶200; Synaptic System, Gottinghem, Germany); guinea pig polyclonal anti-vesicular glutamate transporter-1 (VGLUT1, 1∶2,000; Synaptic System); monoclonal against the calcium-binding protein parvalbumin (PV, 1∶10,000; Swant, Bellinzona, Switzerland), as GABAergic marker. Selected sections were processed for triple-labeling sequential IF with VGLUT1 and VGAT antibodies and a pan-neuronal fluorescent marker (NeuroTrace™, Molecular Probe Inc Eugene OR, USA) to quantify synaptic input. Reacted sections were examined with a Radiance 2100 laser-scanning confocal microscope (Bio-Rad, Hercules, CA, USA) or with Nikon D-Eclipse C1 confocal microscope (Nikon, Tokyo, Japan). To test for antibody specificity, primary antibodies were omitted or replaced with unrelated IgG in control experiments.

### Western blot analysis

Equal amount of proteins from total cortical and hippocampal homogenates were prepared as reported [26]. Protein samples were separated by SDS-PAGE (7% acrylamide), electroblotted onto nitrocellulose, and probed with the following antibodies in 3% non-fat milk: monoclonal anti-NR1 (1∶1,000; Pharmingen, San Diego, CA, USA); monoclonal anti-NR2A (1∶500; Zymed, Carlsbad, CA, USA); polyclonal anti-NR2B (1∶500; Zymed), polyclonal anti-NR2AB (1∶500; Chemicon, International Inc, Temecula, CA, USA), monoclonal anti-CaMKII (1∶2,000; Chemicon); polyclonal anti-phospho-NMDA Receptor 2B (Tyr1472) (1∶1,000; ThermoScientific, Rockford, IL USA); monoclonal anti-PSD95 (1∶1,000; Affinity BioReagents, Golden, CO, USA); monoclonal anti-SAP97 (1∶1,000; Affinity BioReagents) against the synapse associated protein SAP97; polyclonal anti-GluR1 and anti-GluR2/3 (1∶500; Chemicon) against the subunits of the α-amino-3-hydroxy-5-methylisoxazole-4-propionic acid (AMPA) receptor. A monoclonal antibody against actin was used as loading control (1∶3,000; Chemicon).

### Golgi-Cox impregnation

After perfusion, brains were processed for modified Golgi-Cox staining as described by Gibb and Kolb [34]. Brains were first dropped into Golgi-Cox solution (potassium dichromate 5%, mercuric chloride 5% and potassium chromate 5%) and kept in the dark for 14 days at RT, then three days in 30% sucrose at 4°C. Coronal sections of 100 µm thickness were cut with a vibratome (reservoir was filled with 6% sucrose and blade prepared for sectioning by immersion in xylene for 5 min). Slices were immediately collected on 0.5% gelatin-coated microscope slides. Golgi staining was developed in the dark at RT as follows: sections were washed in distilled H_2_O, incubated in ammonium hydroxide (Sigma-Aldrich) for 30 min, dipped in Kodak Fix solution (Rapid fixer; Sigma-Aldrich) for 30 min, washed in distilled H_2_O, dehydrated, cleared and mounted with DPX (BDH Lab Supplies, Leicestershire, UK).

### Dendritic branching and spine density analysis

The Golgi-impregnated neocortical and hippocampal neurons were analyzed at high magnification (100× oil immersion objective) with the Neurolucida software (MicroBrightField Inc., Williston, USA) and a Nikon Eclipse 600 microscope equipped with a motorized stage interfaced to a computer. To select enlarged pyramidal neurons in somatosensory/motor cortex the following criteria were used [35]–[36]: i) characteristic triangular soma shape; ii) full impregnation of the neurons with no apparent dendritic truncation; iii) presence of at least two primary basilar and one apical dendrites, each of which branched at least once, and numerous dendritic spines; iv) soma size ≥300 µm^2^. A total of 31 neurons were fully reconstructed three dimensionally from MP-3m rats and 21 from MDP rats. Dendrites arising from the cell body were considered as first-order until they bifurcated into second-order segments and so on. For analytical purposes, data on apical and basal dendrites were kept separated. The following parameters were quantified for each reconstructed neuron with the software program NeuroExplorer (MicroBrightField Inc.): i) total dendritic length; ii) dendritic branch number and length per order; iii) total dendritic spine number, iv) dendritic spine density, expressed as the ratio number of spines/length of dendritic segment (30 µm), for all dendritic branches, except first order ones.

For each neuron, the dendritic tree complexity was also quantified using Sholl analysis [37] as follows: a transparent grid with concentric rings 10 µm apart was automatically placed by the software over the dendritic drawings and the number of ring intersections was used to estimate the total dendritic length and arborization. The number of dendritic intersections crossing each 10 µm-radius circle progressively more distal from the soma was automatically counted. For each of these parameters, an average was calculated for each animal. Means for MP-3m and MDP-3m groups were then obtained from these individual values and statistically compared by means of t-test.

### Data analysis and statistical evaluation

*Cortical thickness* was measured in 3 serial thionine sections from: i) the motor or *rostral cortex* (0.2/-0.3 mm from bregma), ii) the somatosensory or *heterotopic frontoparietal cortex* (-3.3 mm from bregma), iii) the temporal or *posterior cortex* (-4.8 mm from bregma). Sections were photographed with a Nikon Digital Sight Camera, and the cortical thickness was measured in each section at 0° (1 mm lateral to the midline), 45° and 90° from the midline [26]. The three measures per section were averaged to a single value and the obtained measures from the 3 serial sections from each areas averaged again to a single value to obtain the mean cortical thickness of rostral, somatosensory and posterior cortex for each rat. At least 4 rats from epileptic MP rats and MDP controls at different stages were analyzed (except for MDP-6m, n = 3). Differences among groups were statistically analyzed for each neocortical area by means of one-way analysis of variance (ANOVA) followed by Bonferroni as post-hoc comparison test.

For *three-dimensional hippocampal reconstruction*, thionine stained sections were analyzed with a Nikon Eclipse 600 light microscope equipped with a motorized stage interfaced with a computer. Hippocampal areas were outlined from epileptic MP rats and corresponding controls (at least n = 4 rats/each group) in regularly spaced sections (300 µm) using the Neurolucida software. Total hippocampal volume was obtained using NeuroExplorer software for computer-aided microscopy (MicroBrightField Inc.) [38]. Mean volumes from different groups were compared and statistical analysis performed by means of one-way ANOVA followed by Bonferroni test.

To analyze *granule cell layer (GCL) dispersion*, about 20 consecutive measurements encompassing the lower (infra-pyramidal) GCL were taken at 40–50 µm intervals [39]–[40] in five regularly spaced thionine-stained sections per rat from epileptic MP-3m/MP-6m rats and MDP-3m controls (at least 4 rats/each group). The distance from the inner (hilar) border to the outer border of the most distal granule cell soma was determined using AxioVision software (Carl Zeiss AG, Oberkochen, Germany). All measurements were averaged per section for each rat, compared among groups and statistical analysis was performed by means of one-way ANOVA followed by Bonferroni test.

Quantification of *neurons over-expressing neurofilaments* (NF200^+^ or SMI311^+^) was performed as previously reported [26]. Briefly, 3 sections through the rostral/motor and somatosensory cortex were chosen from each rat (at least 4 rats/each group). Only pyramidal neurons clearly displaying a nucleolus on the plane of the section, with soma size ≥400 µm^2^ were counted by means of AxioVision software in at least 4 adjacent non-overlapping 0.1 mm^2^ subfields per section. Values (n° of cells/area) from rostral and somatosensory cortex were averaged per individual rat, and data from different groups compared and statistically analyzed with one-way ANOVA followed by Bonferroni test. Although our method bears some limitations if compared to stereological analysis, any counting bias should equally affect the diverse samples considered, likely not influencing the final statistical analysis of differences among experimental groups.

*Somatic area and apical dendrite thickness*were evaluated as previously reported [26]. NF200^+^ and SMI311^+^ pyramidal neurons were identified using a Nikon Microphot FXA microscope with Nomarski differential interference contrast at 400× magnification, photographed with a Nikon Coolpix camera and analyzed with the ImageJ software (available via http://rsbweb.nih.gov/ij/). At least 25 neurons/per animal displaying a nucleolus on the plane of the section were analyzed from each group (at least n = 4 rats each group). Mean cell area and apical dendritic thickness (measured at 5 and 15 µm from the upper edge of the nucleus) were averaged for each rat and compared among groups. Statistical analysis was performed by means of one-way ANOVA followed by Bonferroni test.

To *quantify pre-synaptic terminals* at least 3 sections from the frontoparietal cortex (-1.8/-3.8 mm from bregma) were analyzed for each animal. Maximum intensity projections were generated from groups of 4 consecutive optical sections of 0.50 µm z-step to include the Neurotrace^+^ cell body and apical dendrite. Neocortical VGAT^+^ and VGLUT1^+^ pre-synaptic terminals on single enlarged pyramidal neuron were analyzed in high-power (60× objective oil-immersed lens) non-overlapping images from epileptic MP-3m/MP-6m rats and MDP-3m/MDP-6m controls. After background subtraction, the mean intensity fluorescence of peri-somatic/peri-dendritic VGAT^+^ and VGLUT1^+^ terminals were measured in the same region (about 2-3 µm width close to the plasma membrane of soma and apical dendrites; up to 30 µm from the upper edge of the nucleus) using ImageJ software and expressed as VGAT/VGLUT1 immunofluorescence ratio. At least 20 enlarged neocortical neurons (soma size ≥400 µm^2^) for each animal were quantified. The same sections through the neocortex were analyzed to quantify synaptic terminals in the hippocampus. At least three non-overlapping fields from granule cell and CA3 layers were analyzed for each rat. As described for cortical pyramidal neurons, the IF intensity of both VGAT^+^ and VGLUT1^+^ terminals were measured in the same peri-somatic regions of all NeuroTrace^+^ CA3 pyramidal and DG granule cells considered and expressed as VGAT/VGLUT1 ratio. All measurements were averaged for each rat and compared among groups. Statistical analysis was performed by means of one-way ANOVA followed by Bonferroni test. At least six rats from epileptic MP rats and MDP controls at different stages were analyzed (except for MDP-6m, n = 3).

To *quantify western blot data*, primary antibodies were detected with IRDye 680-labeled goat-anti-rabbit IgG or IRDye 800-labeled goat-anti-mouse IgG (LI-COR Biosciences, Lincoln, NE) at 1:10,000 dilution. Bands were visualized and quantified using an Odyssey Infrared Imaging System (LI-COR Biosciences), normalized versus actin signals and compared among groups. Ratio between phospho-NR2B (pNR2B)/total NR2B antibodies was used as measure of the activation of the NR2B-NMDA subunit in each sample. The mean value of MDP group was set at 100 and the single values from experimental rats were expressed as percentage of mean MDP value. Statistical analyses were performed by means of ANOVA followed by Bonferroni test (at least n = 4 rats each group).

The *sample size of rats* necessary to detect a difference of 15% with a power of 80% and alpha 0.05 [41] was estimated using variance values obtained in previous similar morphometric determinations [26] and preliminary analysis. The accuracy of the sample size used was further assessed by using the GraphPad StatMate software (GraphPad Software, Inc. La Jolla, CA USA).

All morphometric measurements were performed independently by two operators blind to the animal treatment. All data were expressed as mean ± SD and differences were considered significant with p<0.05.

## Results

### SE and seizure assessment

Twenty-two out of 83 MP rats (∼25%) did not develop SE and 16 out of 61 rats (∼26%) did not survive SE. As assessed by video-recordings, all MP rats experiencing and surviving SE after pilocarpine developed spontaneous seizures (MP rats, n = 45), in keeping with what previously reported [26]. The onset of the first, video-recorded spontaneous seizure occurred at 7.92±4.46 days after SE induction. Seizure frequency per rat, i.e., the mean number of stage 4-7 seizures/day [30]–[32], was 2.21±0.68 (2.12±0.56 SRS/day in the first week, 2.68±0.64 in the 1^st^-3^rd^ months, and 1.83±0.84 in the 4^th^-6^th^ months of epilepsy). As expected, MDP rats did not develop either behavioral SE or spontaneous seizures.

### Long-term cortical and hippocampal atrophy

**Figure 1:**
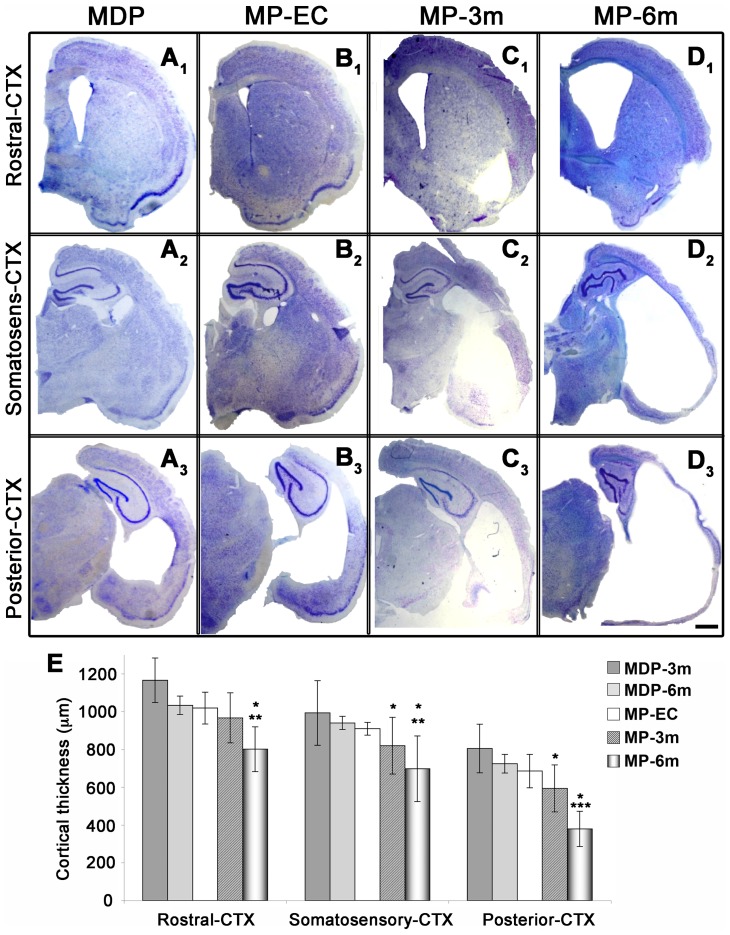
Progressive neocortical atrophy in epileptic MP rats. **A–D**) Low-power thionine-stained coronal sections from rostral (A_1_–D_1_), somatosensory (A_2_–D_2_) and posterior (A_3_–D_3_) cortical areas in representative non-epileptic MDP and epileptic MP rats at different epilepsy stages (EC, early chronic; 3m, 3 months; 6m, 6 months. **E**) Statistical analysis of cortical thickness (at least n = 4 rats each group, except of MDP-6m, n = 3). Note the progressive epilepsy-dependent cortical thinning. Rostral cortex: MP-6m *vs* MDP-3m, MD6m and MP-EC **p<0.01; MP-6m *vs* MP-3m *p<0.05. Somatosensory cortex: MP-6m *vs* MDP-3m, MDP6m and MP-EC **p<0.01; MP-3m *vs* MDP-3m, MDP-6m and MP-EC *p<0.05; MP-6m *vs* MP-3m: *p<0.05. Posterior cortex: MP-6m *vs* MDP-3m, MDP-6m and MP-EC ***p<0.001; MP-3m *vs* MDP-3m, MDP-6m and MP-EC *p<0.05; MP-6m *vs* MP-3m: *p<0.05. Scale bar: 1 mm.

**Figure 2:**
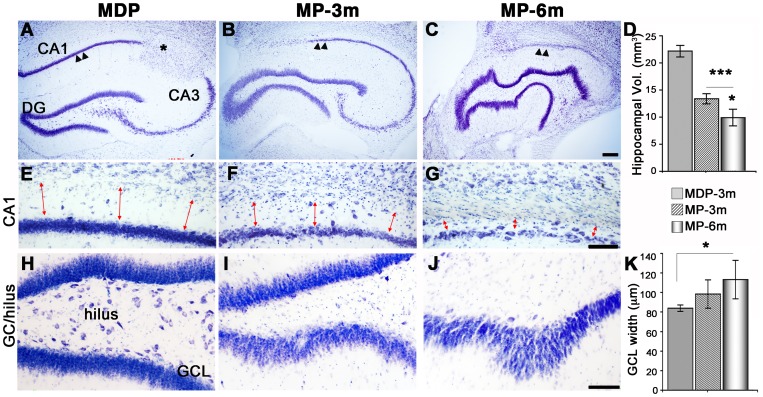
Progressive hippocampal changes in epileptic MP rats. **A–C**) Low-power thionine-stained coronal sections from dorsal hippocampus revealing progressive morphologic changes in chronic epileptic rats. Note the progressive neuronal loss in CA (arrowheads in C *vs* B *vs* A), and the twisted, irregular shape of DG particularly evident in MP-6m rats (C). Asterisk in panel A marks a para-hippocampal heterotopic nodule. **D**) 3-D reconstruction and volumetric analysis of whole hippocampi showed a significant and progressive decrease of hippocampal volume during epilepsy course (MP-3m, 13.40±1.88 mm^3^, and MP-6m, 10.72±2.7 mm^3^, *vs* MDP-3m, 22.17±2.2 mm^3^: ***p<0.001; MP-6m *vs* MP-3m: *p<0.05). **E–G**) The CA1 region was severely affected during epilepsy, as indicated by progressive loss of pyramidal neurons and collapse of the stratum oriens (red arrows). **H–J**) Progressive loss of hilar neurons and increase of GCL thickness in epileptic MP (I–J) *vs* MDP-3m rats (H). **K**) Quantification of lower GCL thickness showed a progressive increase over time (MP-6m *vs* MDP-3m: *p<0.05). Scale bars: 200 µm in A–C; 100 µm in E–J.

We first verified whether cortical atrophy was progressive over time (Figure 1 and Figure 2) by analyzing MP rats at different stages after epilepsy onset (MP-EC, n = 4; MP-3m, n = 6; MP-6m, n = 6) and non-epileptic MDP controls at 3 and 6 months after pilocarpine treatment (MDP-3m, n = 6; MDP-6m, n = 3). As reported in Figure 1, the morphometric analysis of thionine-stained coronal sections from somatosensory/heterotopic (mid-thalamic levels, -3.3/-3.8 mm from bregma) and posterior (posterior thalamic levels, -4.8 mm from bregma) cortical areas showed significant, progressive decrease of cortical thickness that paralleled epilepsy duration. The extent of cortical thinning was steadily progressive in the somatosensory (Figure 1A_2_–D_2_ and E) and dramatic in the posterior cortex (Figure 1A_3_–D_3_ and E). The thickness of rostral/motor cortical areas (anterior commissure level, -0.3 mm from bregma) was only modestly affected after 3 months, but significantly reduced after 6 months of epilepsy (Figure 1A_1_–D_1_ and E). A slight but non-significant decrease was present at the EC stage in all cortical areas considered when compared to corresponding areas of non-epileptic MDP rats (compare B_1_–B_3_ with A_1_–A_3_). The mean cortical thickness in MDP-6m rats was not different from that of MDP-3m rats but it was significantly higher than that of MP-6m rats (Figure 1E and Figure S1). Thus, morphometric data suggest a time-dependent progression of neocortical damage specifically related to SE/subsequent seizures and not to the aging process.

In parallel to neocortical atrophy, we found significant, progressive decrease in the hippocampal volume of epileptic MP rats when compared to MDP-3m controls (Figure 2A–D). However, CA pyramidal neurons and granule cells were differently affected. CA pyramidal neurons were reduced in all epileptic rats, particularly in MP-6m rats with higher seizure frequency (arrowheads in Figure 2A–C). The degree of pyramidal neuron loss was paralleled by reduced thickness of the stratum oriens (red arrows in Figure 2E–G). In contrast, neuronal loss in the dentate gyrus (DG) was mostly restricted to hilar neurons (Figure 2H–J), whereas granule cells became progressively more dispersed (Figure 2H–J and K) giving a twisted and irregular appearance to the granular layer (Figure 2C). The two phenomena were associated, since we observed more evident granular layer abnormalities in MP rats with greater neuronal loss in the CA subfields and hilar region. Thus, a combination of epilepsy-related neuronal loss and *de novo* neurogenesis of granule neurons likely contributed to modify the hippocampal morphology in the course of epilepsy. Hippocampal morphology in MDP-6m rats (Figure S1E) was similar to that of MDP-3m brains, with neither CA loss nor DG abnormalities, indicating that also hippocampal changes in MP-6m were not related to aging or embryonic MAM exposure.

### Pyramidal neurons: Increase in size, dendritic reshaping and spine loss

**Figure 3:**
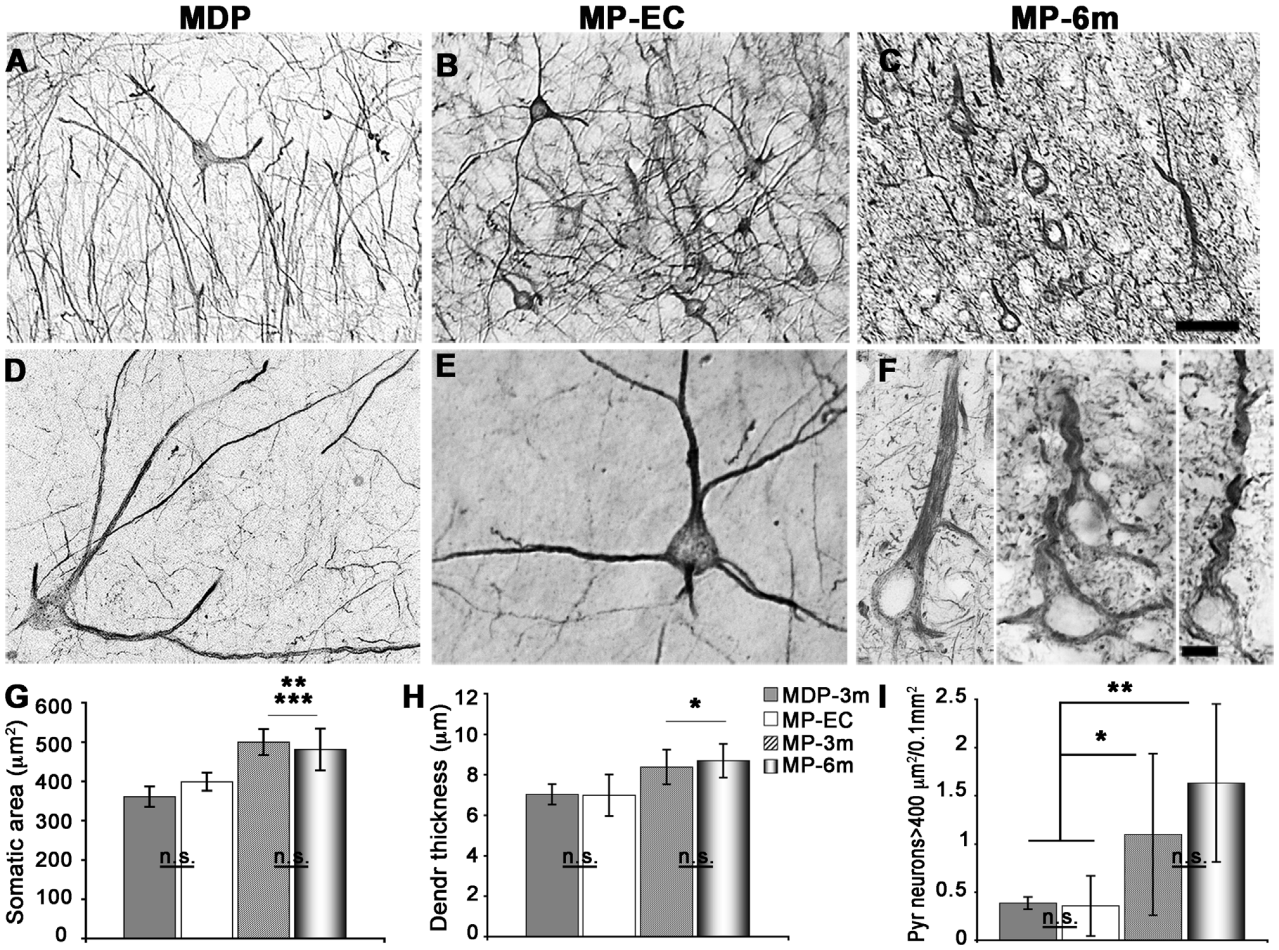
Dysmorphic neocortical pyramidal neurons in epileptic MP rats. **A–F**) Low- (A–C) and high-power (D–F) microphotographs of NF200^+^ enlarged pyramidal neurons in MDP (A, D) and epileptic MP-EC (B, E) and MP-6m (C, F) rats. Note the progressive perikaryal enlargement and dendritic simplification of dysplastic neurons in MP-6m rats *vs* both non-epileptic MDP and early-chronic epileptic MAM rats. **G–I**) Quantification and statistical analysis of somatic area (G), dendritic thickness (H) and cell numbers (I) of NF200^+^ enlarged pyramidal neurons (≥400 µm^2^) at different epilepsy stages in MAM rats (*p<0.05; **p<0.01; ***p<0.001; n.s: not significant). For somatic area and dendrite thickness at least 4 animals/each group were analyzed (25 neurons per animal). Scale bars: 100 µm in A–C; 10 µm in D–F.

Altered neocortical pyramidal neurons were previously reported in MAM-treated rats [27], [42]. A striking feature in the neocortex of epileptic MP rats was the presence of abnormally large pyramidal neurons with neurofilament over-expression and NMDA recruitment to the membrane [26]. We therefore analyzed in the course of epilepsy somatic area, apical dendrite thickness, and numbers of pyramidal neurons with increased expression of neurofilaments (NF200^+^ neurons, Figure 3). Soma size and apical dendrite thickness were significantly increased in MP rats after 3 and 6 months of epilepsy when compared to MDP-3m (***p<0.001 in G, *p<0.05 in H) and MP-EC rats (**p<0.001 in G, *p<0.05 in H), but they did not differ in MP rats after 3 or 6 months of epilepsy. In contrast, the number of NF200^+^ enlarged pyramidal neurons (soma size ≥400 µm^2^) was progressively increased in MP rats during the course of epilepsy. After 6 months of epilepsy, they became more numerous in motor and somatosensory cortical areas (Figure 3I).

**Figure 4:**
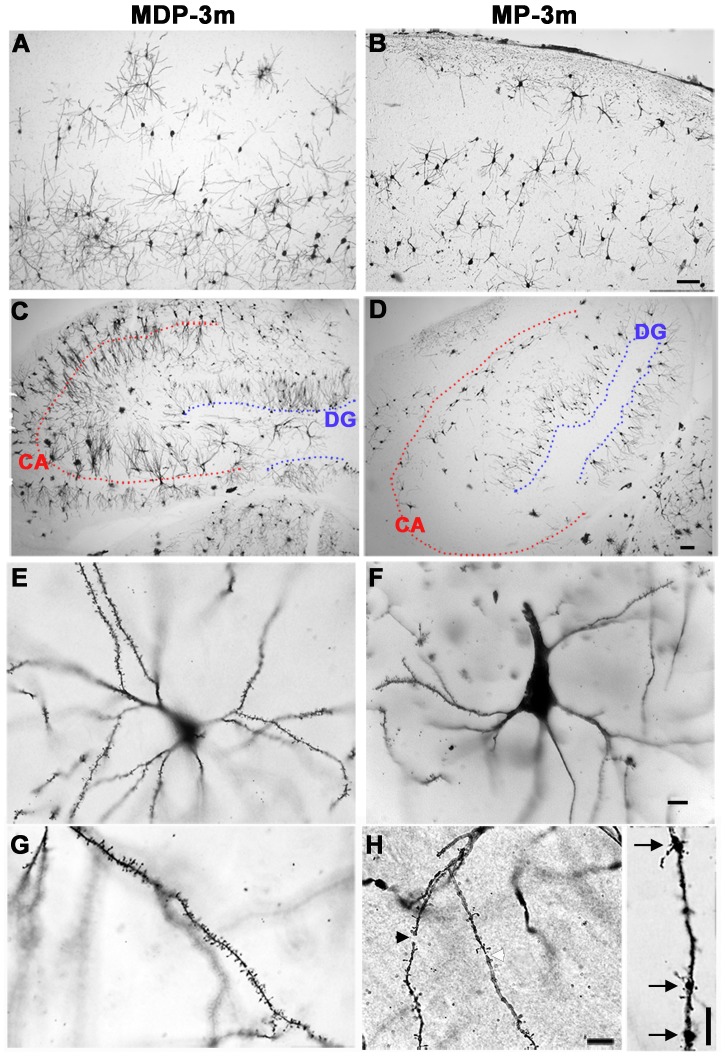
Golgi-Cox analysis of neocortical and hippocampal neurons. A–H) Low- (A–D) and high-power (E–H) microphotographs of Golgiimpregnated neocortical (A–B, E–H) and hippocampal (C–D) neurons from MDP-3m (A, C, E, G) and epileptic MP-3m (B, D, F, H) rats. Note the dramatic and diffuse loss of dendritic arbors (B, D *vs* A, C), increase of both soma size and apical dendrite thickness (F *vs* E), and loss of spines (H *vs* G) in neurons from epileptic MP-3m *vs* non-epileptic MDP-3m rats. Dendrite fragmentations (black arrowhead in H), constrictions (white arrowhead in H) and varicosities (arrows in H, inset) are evident in higher magnification images. Dotted red and blue lines mark CA pyramidal and granule cell layers, respectively (C–D). Scale bars: A–B and C–D, 100 µm; E–H and inset, 10 µm.

The NF200^+^ cortical pyramidal neurons after 3 and 6 months of epilepsy showed simplified dendritic arbors when compared to MP-EC and MDP rats (Figure 3, compare F with D–E). Since modifications of dendritic morphology may reflect changes in synaptic input, we examined dendritic branching and spine density in Golgi-Cox stained sections from somatosensory cortex and hippocampus of 5 MP-3m *vs* 4 MDP-3m rats. As reported in Figure 4, the Golgi-Cox labeling confirmed in both neocortical (Figure 4A–B) and hippocampal (Figure 4C–D) neurons an impressive loss of dendritic branching in MP-3m *vs* MDP-3m. The decreased arborization of basal and apical dendrites was evident in neocortical and hippocampal pyramidal neurons and in granule cells even at low-power magnification (Figure 4B, D *vs* A, C). At higher magnification, dendritic abnormalities of MP rats became more evident: reduced branching (F *vs* E), spine loss (H *vs* G), and dendritic fragmentations (black arrowhead in H), constrictions (white arrowhead in H), and varicosities (arrows in H, inset).

**Figure 5:**
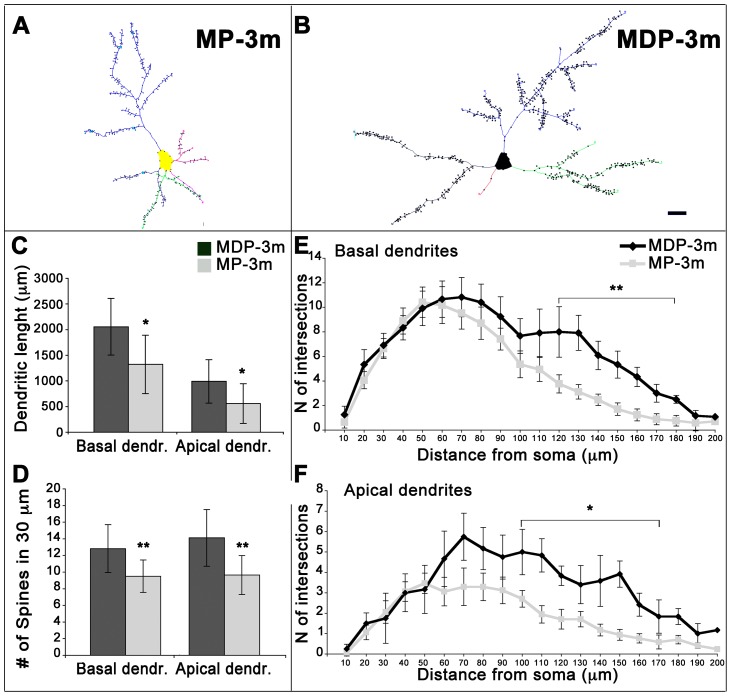
Quantification of dendritic branching and spine density in epileptic MP rats. **A–B**) Neurolucida tracings of representative Golgi-impregnated cortical pyramidal neurons from chronic epileptic MP-3m (A) and non-epileptic MDP-3m (B) rats. Note the clear reduction of dendritic tree complexity and spine density in the MP neuron in panel A. Scale bar: 20 µm. **C–D**) Quantitative analysis of total dendrite length (C) and spine density (D) of basal and apical dendrites from fully reconstructed cortical pyramidal neurons in MDP-3m *vs* MP-3m (*p<0.05; **p<0.01). **E–F**) Sholl dendrite analysis, obtained by placing a series of concentric circles spaced at 10 µm intervals centered on the soma, demonstrated a significant reduction in dendritic arborization of both basal (**p<0.01) and apical (*p<0.05) dendrites in MP-3m *vs* MDP-3m rats.

We then quantified dendritic length, complexity, and spine density in neocortical pyramidal neurons (see Figure 5). Tracings of representative fully reconstructed neurons from chronic epileptic MP-3m rats and MDP-3m controls are reported in Figure 5A–B. The average total dendrite length of both apical and basal dendrites of cortical pyramidal neurons was significantly lower in MP than corresponding MDP neurons (Figure 5C, *p<0.05). Spine density was significantly decreased both in basal and apical dendrites of pyramidal neurons in MP compared to MDP rats (Figure 5D, **p<0.01). The analysis of dendritic complexity (mean dendrite number and length per branch order) showed reduced arbor branches and shortening of dendritic segments both in apical and basal dendrites of MP cortical pyramidal neurons, the most distal dendrites (from the fourth to seventh branch orders) being particularly affected (data not shown). Dendrite complexity was further assessed by Sholl analysis (Figure 5E–F). Significantly reduced intersections between dendrites and Sholl circles occurred at a distance of 100–120 to 170–180 µm from the soma in both basal (Figure 5E, **p<0.01) and apical dendrites (Figure 5F, *p<0.05) of pyramidal neurons.

### Synaptic abnormalities

**Figure 6:**
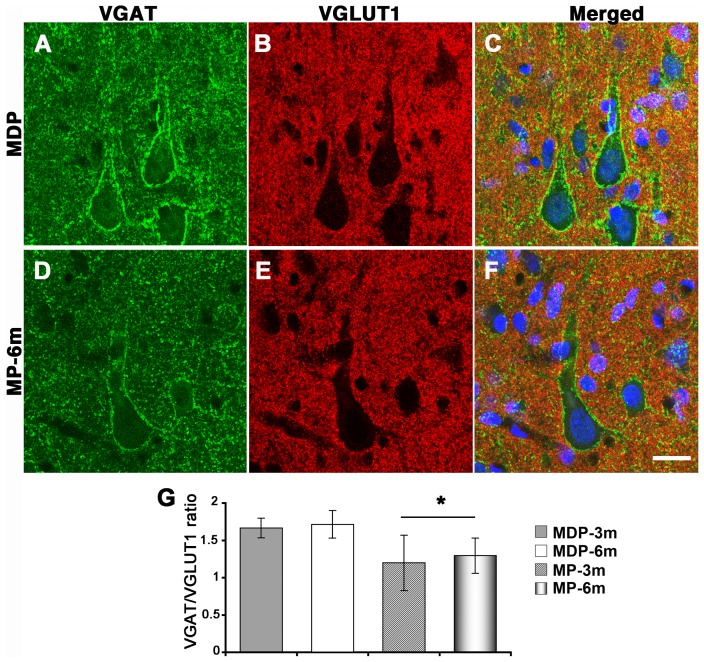
Altered synaptic input on neocortical pyramidal neurons of epileptic MP rats. **A–F**) VGAT^+^ (green) and VGLUT1^+^ (red) synaptic terminals on pyramidal neurons from non-epileptic MDP (A–C) and epileptic MP-6m rats (D–F). Panels C and F are merged images counterstained with Neurotrace™ (blue) to reveal neuronal nuclei. **G**) Quantification of VGAT^+^ and VGLUT1^+^ peri-somatic and -dendritic terminals on neocortical pyramidal neurons expressed as VGAT/VGLUT1 IF ratio. VGAT/VGLUT1 ratio was significantly decreased in MP *vs* aged-matched MDP control (*p<0.05 MP-3m/6m *vs* MDP-3m/6m). No differences were found in MDP-3m *vs* MDP-6m and MP-3m *vs* MP-6m. Scale bar: 20 µm.

**Figure 7:**
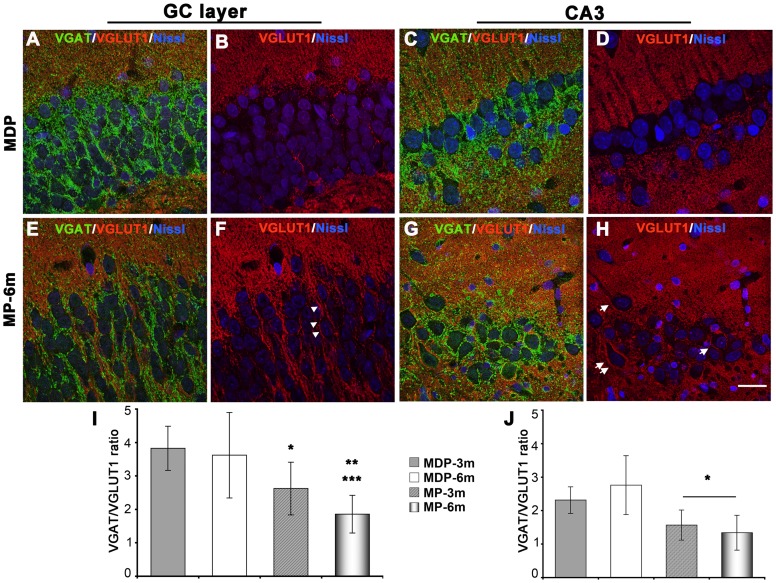
Altered synaptic input on hippocampal neurons of epileptic MP rats. **A–H**) VGAT^+^ (green) and VGLUT1^+^ (red) synaptic terminals on granule cells (A–B and E–F) and CA3 pyramidal neurons (C–D and G–H) from a representative non-epileptic MDP (A–D) and MP-6m rats (E–H). Sections were counterstained with Neurotrace™ (blue) to reveal neuronal nuclei. Note in MDP rats the intense VGAT^+^ staining outlining cell bodies (A and C, green) and the virtual absence of VGLUT1^+^ staining in both GC and CA3 layers (B and D, red). In MP-6m rats, by contrast, perisomatic VGAT^+^ staining was reduced (compare E *vs* A, G *vs* C) but VGLUT1^+^ synaptic terminals were more evident in both GC and CA3 layers (compare F *vs* B, H *vs* D), sometimes outlining neuronal profiles (arrowheads in F, arrows in H). **I–J**) Quantification of VGAT/VGLUT1 IF ratio at peri-somatic and -dendritic terminals on granule cells (I) and CA3 pyramidal neurons (J). Note the significant reduction VGAT/VGLUT1 ratio in both layers in MP rats compared to aged-matched MDP controls (**I**: MP-6m *vs* MDP-3m ***p<0.001; MP-6m *vs* MDP-6m **p<0.01; MP-3m *vs* MDP3m/MDP-6m *p<0.05; **J**: MP-3m/MP-6m *vs* MDP-3m/MDP-6m rats, *p<0.05). No differences were found in MDP-3m *vs* MDP-6m and MP-3m *vs* MP-6m. Scale bar: 20 µm.

The evident dendritic abnormalities of neocortical and hippocampal neurons prompted us to analyze synaptic input in both cortical areas. We evaluated excitatory and inhibitory input to soma and proximal dendrites of enlarged neocortical pyramidal neurons (soma size ≥400 µm^2^), CA3 pyramidal neurons and granule cells by means of confocal IF in sections reacted with specific GABAergic (VGAT) and glutamatergic (VGLUT1) synaptic markers (Figures 6, 7, and S2-4). MP-3m and MP-6m rats were analyzed and compared to corresponding MDP controls. VGAT staining was decreased in cortical pyramidal neurons (Figure 6A *vs* D, Figure S2 A *vs* B, C *vs* D), granule cells of the dentate gyrus (Figure 7A *vs* E, Figure S3 A *vs* B, C *vs* D) and CA3 pyramidal neurons (Figure 7C *vs* G, Figure S4 A *vs* B, C *vs* D) of chronic epileptic MP-3m/MP-6m rats when compared to corresponding MDP controls. Reduction in inhibitory input was also confirmed by the overall reduction of PV puncta in the same areas (data not shown). By contrast, VGLUT1 synaptic terminals were more evident around hippocampal granule cells and CA3 pyramidal neurons of MP rats (Figure 7B *vs* F, D *vs* H; S3 and S4, E *vs* F, G *vs* H), sometimes clearly outlining perikaryal profiles (arrowheads in Figure 7F, arrows in 7H). The ImageJ quantification of peri-somatic and -dendritic labeling revealed significantly reduced VGAT/VGLUT1 ratio on enlarged neocortical pyramidal neurons (Figure 6G), granule cells (Figure 7I) and CA3 pyramidal neurons (Figure 7J) of epileptic MP-3m/MP-6m rats *vs* corresponding MDP controls, thus indicating an overall reorganization of glutamatergic and GABAergic networks in both neocortex and hippocampus of epileptic MP rats. No significant differences in VGAT/VGLUT1 ratio were observed in MP-3m *vs* MP-6m or in MDP-3m *vs* MDP-6m rats.

### NMDA receptor activation

**Figure 8:**
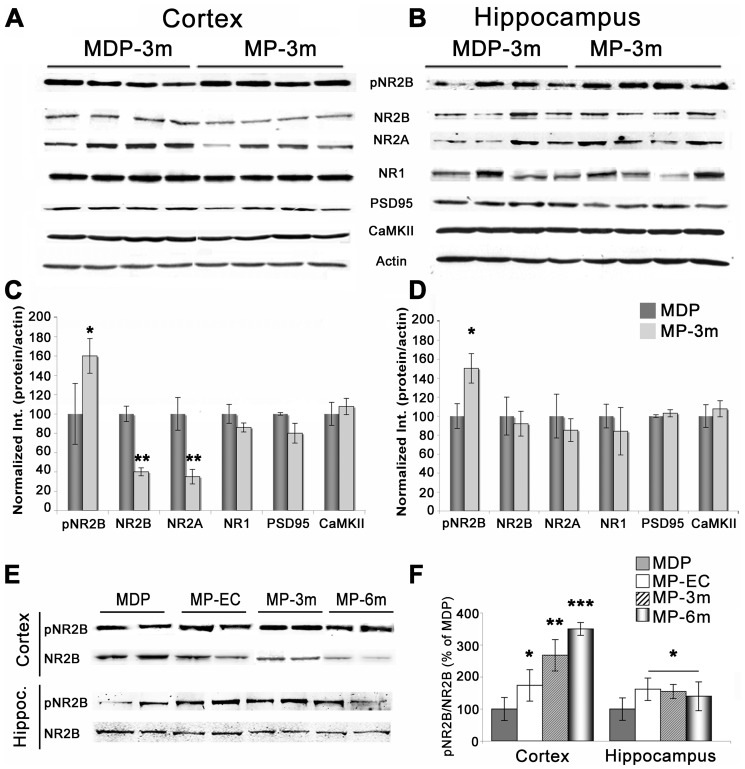
Molecular composition of the glutamatergic synapse in epileptic MP rats. **A–B**) Representative WB analysis in neocortical (A) and hippocampal (B) homogenates from 4 MDP-3m and 4 epileptic MP-3m rats. Note the increased expression of the activated, phospho-tyr-1472-NR2B (pNR2B) subunit in both neocortex and hippocampus in MP-3m *vs* MDP-3m rats, which was associated in the neocortex with decreased NR2A/B subunits. **C–D**) Quantification of NMDA receptor subunits and associated proteins/actin ratio in epileptic MP-3m rats, expressed as percentage of values *vs* non-epileptic MDP rats. Significant increase of pNR2B in neocortex and hippocampus (*p<0.05) and decrease of NR2A/B (**p<0.01) in neocortex only were found in epileptic MP-3m *vs* MDP-3m rats (at least n = 4 rats each group). **E**) Representative WB of pNR2B and total NR2B expression from neocortical and hippocampal homogenates of MP rats at different epilepsy stages and non-epileptic MDP rats. Note the persistent NR2B phosphorylation in both neocortex and hippocampus of MP rats. **F**) Quantification of the pNR2B/total NR2B ratio. The ratio was significantly increased in the hippocampus in early chronic and chronic epilepsy stages (MP *vs* MDP rat *p<0.05) and it increased progressively in the neocortex during epilepsy course (MP *vs* MDP rat *p<0.05, **p<0.01; ***p<0.001; at least n = 4 rats each group).

Finally, to verify the molecular composition of the glutamatergic synapse, we analyzed by means of WB AMPA and NMDA receptors and associated post-synaptic proteins in both neocortical and hippocampal homogenates from epileptic MP rats and corresponding MDP controls. As reported in Figure 8, the NMDA subunits NR2A and 2B were significantly reduced in the neocortex (Figure 8A and C) but not in the hippocampus (Figure 8B and D) of chronic epileptic MP-3m rats, whereas the NR1 subunit, PSD-95, CaMKII (Figure 8A-D), SAP97, GluR1, and GluR2-3 (not shown) were not modified. In addition, phospho-NR2B (pNR2B) levels were significantly higher in both the neocortex (Figure 8C) and hippocampus (Figure 8D) of epileptic MP-3m rats *vs* MDP-3m controls. The phospho-NR2B/total NR2B ratio was increased in both cerebral areas, indicating a persistent hyper-activation of the NR2B subunit in epileptic MP brains. Such NR2B hyper-activation was confirmed at different time-points after epilepsy onset (Figure 8E–F): the phospho-NR2B/total NR2B ratio was consistently and significantly increased in the hippocampus in early chronic and chronic epilepsy stages, and it increased progressively in the neocortex during epilepsy course (Figure 8F), suggesting that steady NR2B activation was a key point in the spontaneous seizure activity of epileptic MP rats.

## Discussion

We previously demonstrated that in the malformed brain of MAM-treated rats the occurrence of pilocarpine-induced SE and subsequent seizures generated a pathologic process capable of modifying cell morphology and NMDA receptor expression in neocortical pyramidal neurons [26]. We have here extended the morphologic and molecular analysis of MAM-pilocarpine rats to both neocortex and hippocampus from few days after epilepsy onset up to six months of recurring seizures to verify whether pathologic brain changes were widespread and progressive over time. Our extended analysis revealed a steadily progressive process set in motion by SE/chronic seizures in the malformed brain, capable of altering dramatically the gross morphology and the fine neuronal structure of both neocortex and hippocampus, changing synaptic terminals favoring excitatory ones, and modifying the glutamatergic synapse, permanently activating NMDA NR2B subunit.

### Progression of brain damage

The deleterious effect of SE and the progression of brain damage along the course of SE-induced seizures were already analyzed in different epilepsy models. It is well-established that the induction of SE can readily damage the brain [5], [43]. However, previous papers failed to demonstrate epilepsy-related damage progression [5] or showed that SE-related damage preceded epilepsy onset [22]. A recent paper demonstrating progressive brain damage did not find a clear correlation with seizure activity [44]. On the other hand, another study demonstrating worsening of seizure frequency after kainate-induced SE did not report whether brain damage paralleled seizure progression [45].

Our data demonstrate that seizure onset can precede macroscopic brain damage, since early chronic epileptic MP rats do not show significant gross anatomical atrophy when compared to non-epileptic MDP controls. More importantly, our data clearly show that SE can trigger a progressive pathologic process unrelated to aging or embryonic MAM exposure. Indeed, some of the abnormal features here reported, like the convoluted appearance of granule cell layer in hippocampus and the presence of abnormally large neurons in neocortex, become evident or more conspicuous at later epilepsy time-points, and were not present in control MDP rats not experiencing behavioral SE/seizures. Since these data do not allow dissecting the contribution of repeated seizures in the pathologic process triggered by SE, we are currently analyzing MAM/pilocarpine rats in which seizures are pharmacologically prevented after the induction of SE to explore such contribution. We believe, however, that SE and subsequent seizures are similar biological phenomena of different severity/semiology [46]. The important point here is the demonstration that abnormal electrophysiologic activities (i.e., SE and subsequent seizures) when sustained, when repeated, are indeed capable of modifying progressively the cerebral structure, thus supporting the view that epileptogenesis is a process starting with SE and extending long beyond the first seizure [45], [47].

### Morphologic and synaptic changes

Many of the morphologic and molecular changes here reported, particularly those in the hippocampus, were previously analyzed in different experimental models. In general, they were described as related to the time of SE induction. Loss of GABAergic interneurons in hilus and stratum oriens, associated with variable loss of pyramidal neurons and granule cells were reported as an early, SE-related event, not extending to later time points [22], [48]–[49]. Hippocampal reorganization, particularly mossy fiber sprouting, was also consistently reported, resulting in subsequent hyperexcitation according to some studies [50]–[51] or in GABA-mediated circuit stabilization according to others [52]–[53]. Enhanced granule cell neurogenesis was temporally related to SE [54]–[55] but also promoted by repeated spontaneous brief seizures in absence of SE [56]. Newly generated neurons not only influenced the reorganization of hippocampal network [55] but they were also deeply affected by the pathologic conditions created by SE or repeated seizures [57]–[59]. VGLUT1 and VGAT staining demonstrated either increased GABAergic and glutamatergic input in the hippocampus after pilocarpine SE [60], or reduced GABA and increased glutamate synaptic input in the neocortex of the irradiated model of cortical dysplasia [25]. Finally, dendritic damage and reshaping and spine loss shortly following SE and seizures was demonstrated in different SE models of experimental epilepsy [22], [61]–[63].

Our data reveal two relevant novel findings. First, they show that the abnormalities induced by the occurrence of SE in the malformed brain are long-term, ongoing brain changes, as demonstrated by the progressive granule cell dispersion, neocortical and hippocampal atrophy, and VGAT/VGLUT1 staining changes in the late chronic epilepsy phase. Second, they indicate a common, widespread pathologic process taking place in the malformed brain. The similar atrophy, dendritic abnormalities, changes in glutamatergic and GABAergic terminals, and NMDA NR2B phosphorylation in both neocortex and hippocampus altogether indicate the existence of a SE and seizure-related, common pathologic process that, once started, is capable of diffusely affecting the epileptic malformed brain.

In addition, non-neuronal mechanisms might also contribute to the morpho-molecular changes here reported. Prenatal MAM can affect vasculogenesis [64] and modify hippocampal morphology if co-administered with the angiogenesis inhibitor thalidomide [65]. Pilocarpine-induced seizures in MAM-treated rats can further exacerbate the intrinsic vascular abnormalities (i.e., blood-brain barrier leakage and abnormal vascular supply) [66], which in turn could produce anoxic cell death thus modifying brain morphology.

### Role of NMDA receptors

What are the molecular mechanisms possibly fostering the progressive changes here reported? Obviously, many different factors are potentially involved. The present data suggest that glutamate could play a role in determining the pathologic plasticity through the activation of NMDA receptors. The relevance of the NR2B subunit in cortical dysplasia was suggested not only by the increased NR2B expression [15], [19] but also by the efficacy of NR2B selective antagonists in blocking epileptiform activities in both experimental models and patients [20], [67]–[68]. In different experimental models of epilepsy, tyrosine phosphorylation of NR2B after SE and spontaneous seizures was short-lasting and therefore the effect and not the cause of seizures [69]–[72]. It is however also possible that enhanced NR2B phosphorylation can directly influence seizure generation by increasing NMDA-dependent Ca^++^ influx [73]–[74]. The stable or even progressive NR2B phosphorylation reported here might suggest an upstream role of NMDA in the seizure-related plasticity, possibly through the activation of downstream signaling pathways leading to cell changes in the postsynaptic neurons [75]–[76] or cell death particularly if extrasynaptic NMDA receptors are involved [69], [77]. The NR2B hyper-activation is a molecular event preceding brain damage, as already present in the early stage of epilepsy (in MP-EC rats), when cellular, cortical and hippocampal abnormalities are not fully established. The demonstration of a primary role of NR2B would, therefore, support the use of drugs specifically acting on NMDA and blocking the activation of NMDA-related death pathways [78]–[79]. Obviously, we demonstrated here expression and phosphorylation state, and not physiology, of NMDA receptors. Additional physiological investigation will be therefore required to fully verify in our model the functional relevance of glutamate hyper-activation and GABA down-regulation.

### Relation to human epilepsy

Are there relationships between what we report here in epileptic MP rats and the situation in human patients? Some Authors criticized the experimental models based on the induction of prolonged convulsive seizures given the rare occurrence of SE as precipitating factor in human epilepsy and the limited extent of hippocampal *vs* extra-hippocampal damage [80]. However, many features reported here in the MAM/pilocarpine convulsive model were also reported in human epilepsy. Hypertrophic neocortical pyramidal neurons with abnormal dendrites and reduced spines, dysmorphic hippocampal neurons and granule cell dispersion in the dentate gyrus were all described in TLE patients [39], [81]–[82]. Shrinkage of the dendritic tree, spine loss, and dendritic swellings here demonstrated in neocortical and hippocampal neurons were equally described in hippocampal pyramidal neurons and granule cells of TLE patients [83]. Interestingly, only neurons with altered dendritic morphology displayed NMDA mediated abnormal firing behavior [84]. Increased VGLUT1 synaptic input was reported in human TLE, particularly prominent in the dentate gyrus of patients with hippocampal sclerosis [85]. Altogether this evidence suggests the progressive nature of severe seizures in both experimental models and humans [1]–[2]. In line with this hypothesis, we have very recently demonstrated that epilepsy duration was positively correlated with morphometric changes of both neurons and glia and greater glutamatergic input in the epileptogenic/dysplastic areas of FCD patients [86]. It is possible, therefore, that the progressive pathologic plasticity we describe here might also occur in severe, malformation-related human epilepsies.

## Supporting Information

Figure S1 **Brain morphology of non-epileptic MDP-6m rats.** Low- (**A–D**) and higher-power (**E**) microphotographs of thionine-stained coronal sections from rostral (A), somatosensory (B, C), posterior (D) cortical areas and dorsal hippocampus (E) of non-epileptic MAM rats after 6 months from pilocarpine treatment. No age-related changes of cortical thickness, CA neuronal loss or DG abnormalities were evident in non-epileptic rats after long time intervals from pilocarpine treatment. Scale bars: 2 mm in A–D, 200 µm in E.
(TIF)

Figure S2 **Synaptic input changes on neocortical pyramidal neurons of non-epileptic MDP** ***vs*** **epileptic MP rats.** VGAT^+^ (**A**–**D**, green) and VGLUT1^+^ (**E**–**H**, red) synaptic terminals on cortical pyramidal neurons from non-epileptic MDP-3m (A, E), MDP-6m (C, G) and epileptic MP-3m (B, F), MP-6m rats (D, H). Note the reduced VGAT^+^ (A *vs* B, C *vs* D) and the slightly more evident VGLUT1^+^ peri-somatic and -dendritic labeling (E *vs* F, G *vs* H) of pyramidal neurons from chronic epileptic MP-3m (B and F) and MP-6m rats (D and H) when compared to corresponding MDP controls (A and E; C and G). Scale bar: 25 µm.
(TIF)

Figure S3 **Synaptic input changes on hippocampal granule cells of non-epileptic MDP** ***vs*** **epileptic MP rats.** VGAT^+^ (**A**–**D**, green) and VGLUT1^+^ (**E**–**H**, red) synaptic terminals on hippocampal GCs from non-epileptic MDP-3m (A, E), MDP-6m (C, G) and epileptic MP-3m (B, F), MP-6m rats (D, H). Note the reduced VGAT^+^ (A *vs* B, C *vs* D) and the more evident VGLUT1^+^ peri-somatic and -dendritic labeling (E *vs* F, G *vs* H) of GCs from chronic epileptic MP-3m (B and F) and MP-6m rats (D and H) when compared to corresponding MDP controls (A and E; C and G). Scale bar: 25 µm.
(TIF)

Figure S4 **Synaptic input changes on CA3 neurons of non-epileptic MDP** ***vs*** **epileptic MP rats.** VGAT^+^ (**A**–**D**, green) and VGLUT1^+^ (**E**–**H**, red) synaptic terminals on hippocampal CA3 pyramidal neurons from non-epileptic MDP-3m (A, E), MDP-6m (C, G) and epileptic MP-3m (B, F), MP-6m rats (D, H). Note the reduced VGAT^+^ (A *vs* B, C *vs* D) and the more evident VGLUT1^+^ peri-somatic and -dendritic labeling (E *vs* F, G *vs* H) of CA3 neurons from chronic epileptic MP-3m (B and F) and MP-6m rats (D and H) when compared to corresponding MDP controls (A and E; C and G). Scale bar: 25 µm.
(TIF)
