# Supplementary figures and images for: Progressive Brain Damage, Synaptic Reorganization and NMDA Activation in a Model of Epileptogenic Cortical Dysplasia
Source: PLoS One. 2014 Feb 27;9(2):e89898. doi: 10.1371/journal.pone.0089898 (PMC3937400; doi:10.1371/journal.pone.0089898)

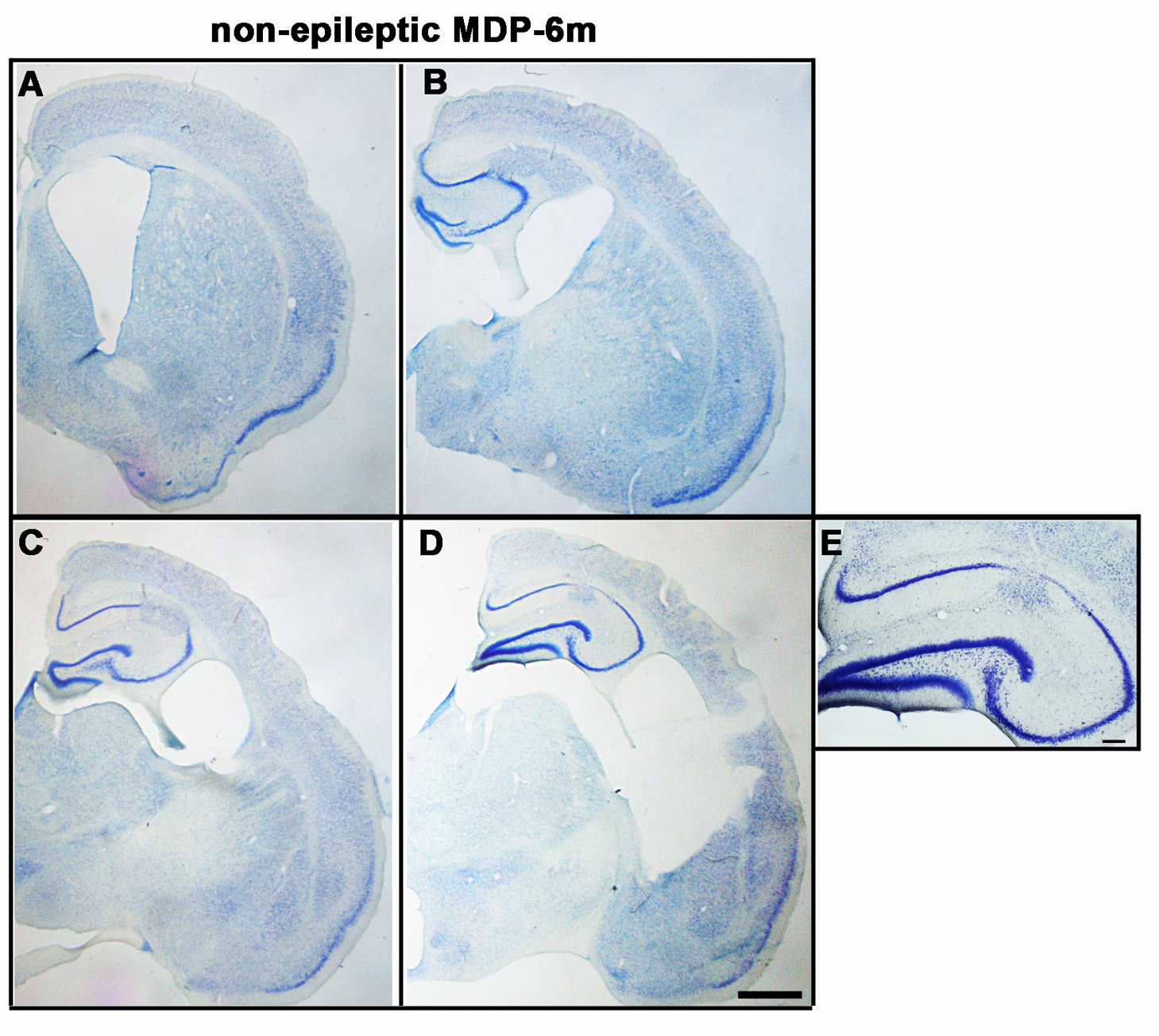

Supplement: Figure S1 — Brain morphology of non-epileptic MDP-6m rats. Low- (A–D) and higher-power (E) microphotographs of thionine-stained coronal sections from rostral (A), somatosensory (B, C), posterior (D) cortical areas and dorsal hippocampus (E) of non-epileptic MAM rats after 6 months from pilocarpine treatment. No age-related changes of cortical thickness, CA neuronal loss or DG abnormalities were evident in non-epileptic rats after long time intervals from pilocarpine treatment. Scale bars: 2 mm in A–D, 200 µm in E. (TIF) [file pone.0089898.s001.tif]

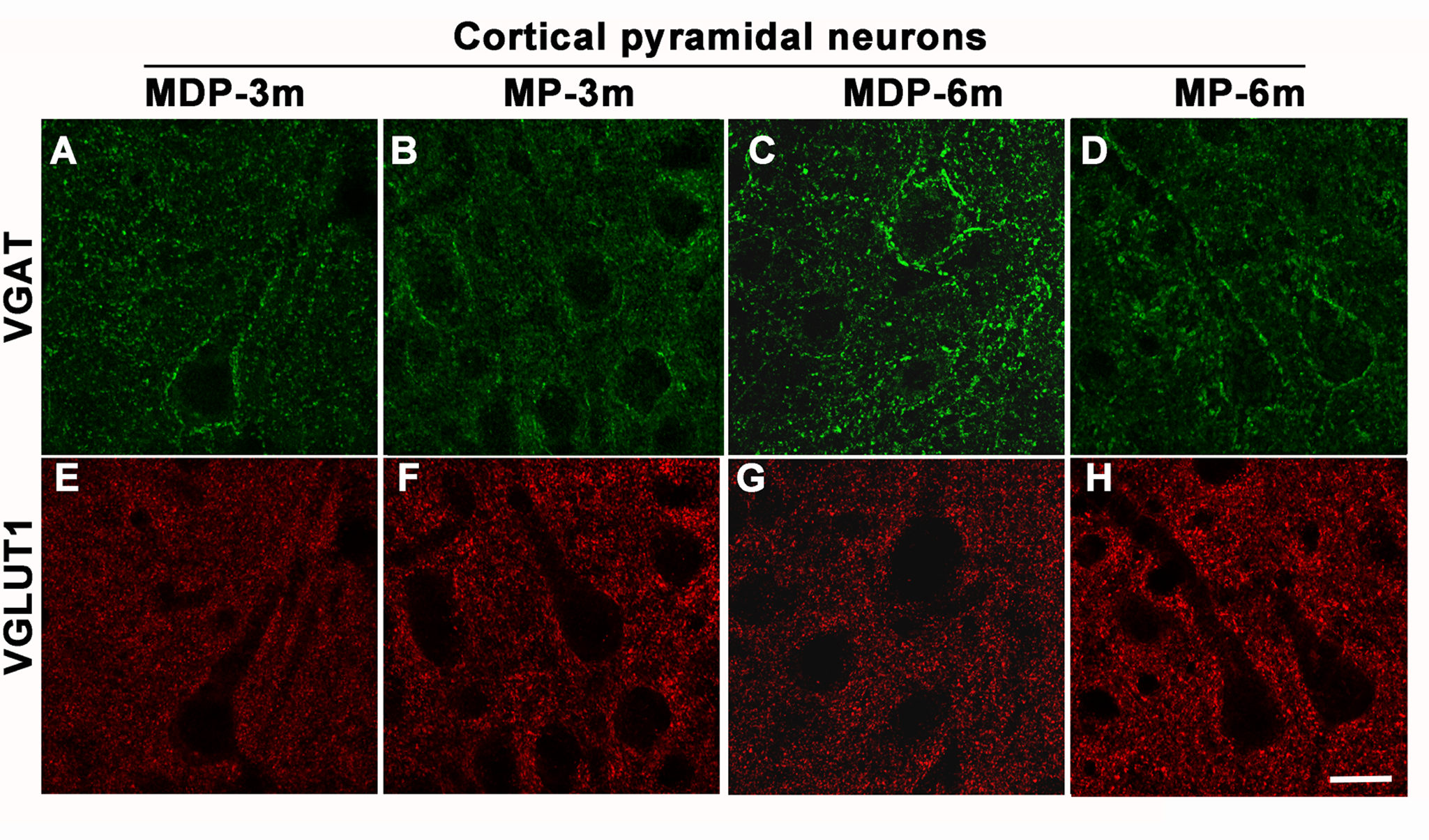

Supplement: Figure S2 — Synaptic input changes on neocortical pyramidal neurons of non-epileptic MDP vs epileptic MP rats. VGAT+ (A–D, green) and VGLUT1+ (E–H, red) synaptic terminals on cortical pyramidal neurons from non-epileptic MDP-3m (A, E), MDP-6m (C, G) and epileptic MP-3m (B, F), MP-6m rats (D, H). Note the reduced VGAT+ (A vs B, C vs D) and the slightly more evident VGLUT1+ peri-somatic and -dendritic labeling (E vs F, G vs H) of pyramidal neurons from chronic epileptic MP-3m (B and F) and MP-6m rats (D and H) when compared to corresponding MDP controls (A and E; C and G). Scale bar: 25 µm. (TIF) [file pone.0089898.s002.tif]

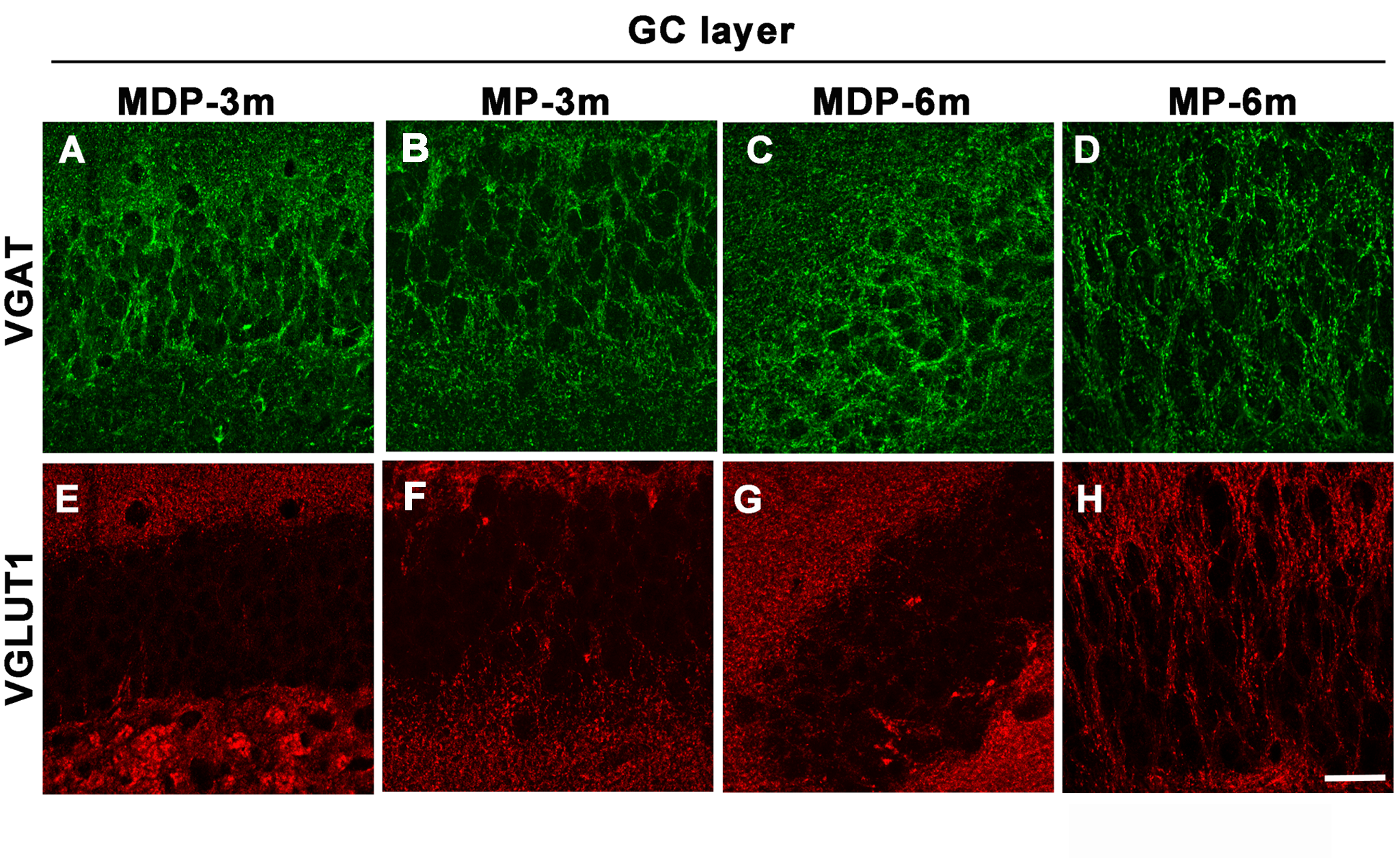

Supplement: Figure S3 — Synaptic input changes on hippocampal granule cells of non-epileptic MDP vs epileptic MP rats. VGAT+ (A–D, green) and VGLUT1+ (E–H, red) synaptic terminals on hippocampal GCs from non-epileptic MDP-3m (A, E), MDP-6m (C, G) and epileptic MP-3m (B, F), MP-6m rats (D, H). Note the reduced VGAT+ (A vs B, C vs D) and the more evident VGLUT1+ peri-somatic and -dendritic labeling (E vs F, G vs H) of GCs from chronic epileptic MP-3m (B and F) and MP-6m rats (D and H) when compared to corresponding MDP controls (A and E; C and G). Scale bar: 25 µm. (TIF) [file pone.0089898.s003.tif]

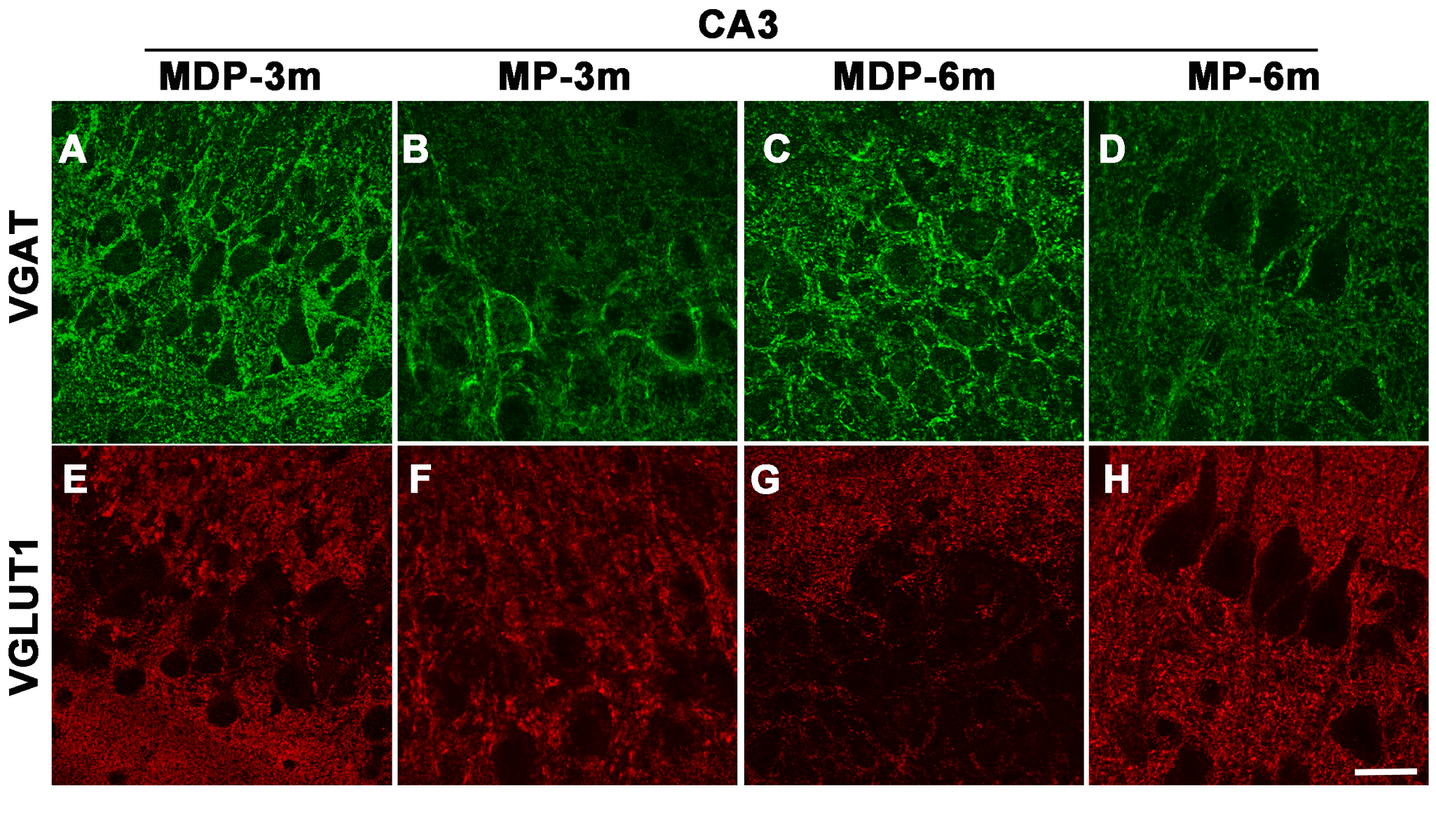

Supplement: Figure S4 — Synaptic input changes on CA3 neurons of non-epileptic MDP vs epileptic MP rats. VGAT+ (A–D, green) and VGLUT1+ (E–H, red) synaptic terminals on hippocampal CA3 pyramidal neurons from non-epileptic MDP-3m (A, E), MDP-6m (C, G) and epileptic MP-3m (B, F), MP-6m rats (D, H). Note the reduced VGAT+ (A vs B, C vs D) and the more evident VGLUT1+ peri-somatic and -dendritic labeling (E vs F, G vs H) of CA3 neurons from chronic epileptic MP-3m (B and F) and MP-6m rats (D and H) when compared to corresponding MDP controls (A and E; C and G). Scale bar: 25 µm. (TIF) [file pone.0089898.s004.tif]
